# A Study of Efficiency Droop Phenomenon in GaN-Based Laser Diodes before Lasing

**DOI:** 10.3390/ma10050482

**Published:** 2017-04-30

**Authors:** Mei-Xin Feng, Qian Sun, Jian-Ping Liu, Zeng-Cheng Li, Yu Zhou, Hong-Wei Gao, Shu-Ming Zhang, Hui Yang

**Affiliations:** 1 The Key Laboratory of Nanodevices and Applications, Chinese Academy of Sciences, Suzhou 215123, China; mxfeng2011@sinano.ac.cn (M.-X.F.); zcli2011@sinano.ac.cn (Z.-C.L.); yzhou2008@sinano.ac.cn (Y.Z.); hwgao2013@sinano.ac.cn (H.-W.G.); smzhang2010@sinano.ac.cn (S.-M.Z.); hyang2006@sinano.ac.cn (H.Y.); 2 Suzhou Institute of Nano-Tech and Nano-Bionics, Chinese Academy of Sciences, Suzhou 215123, China

**Keywords:** laser diodes, efficiency droop, stimulated emission, Auger recombination

## Abstract

Carrier recombination behavior in *c*-plane GaN-based laser diodes (LDs) is numerically investigated by using the commercial software *LASTIP*. It is found that efficiency droop phenomenon does exist in GaN-based LDs before lasing, which is confirmed by experimental results. However, the current density corresponding to the peak efficiency of GaN-based LDs before lasing, *J_max_*, is nearly 40 A/cm^2^, which is much lower than that reported by other studies. The reported *J_max_*, measured from the cavity facet side is modulated by the absorption of quantum wells, which shifts the *J_max_* to a higher value. In addition, the currents due to various recombinations are calculated. It is found that Auger recombination affects the threshold current greatly, but it only plays a small role at high current injection levels.

## 1. Introduction

Efficiency droop phenomenon has been widely observed in III-nitride light-emitting diodes (LEDs) [1,2], which means that the internal quantum efficiency (IQE) of GaN-based LEDs increases with the injection current until it comes to the maximum, afterwards it drops steadily. Normally, the current density corresponding to the peak IQE, *J_max_*, is less than 10 A/cm^2^ [3]. GaN-based laser diodes (LDs) operate like LEDs before lasing and their efficiency droop phenomenon has also been studied [4,5,6]. However, Lutgen et al. reported that the current density corresponding to the peak efficiency of GaN-based LDs before lasing, *J_max_*, is 133 A/cm^2^ [6], which is much larger than that of typical GaN-based LEDs. Understanding the reason behind this could be helpful in studying the origin of efficiency droop and may provide a possible way to alleviate efficiency droop in GaN-based LDs. Nevertheless, few studies have talked about this.

Auger recombination is supposed to be one possible origin of efficiency droop [7,8,9]. Iveland et al. reported on the unambiguous detection of Auger electrons by electron emission spectroscopy from a InGaN/GaN LED under electrical injection, which confirms the existence of Auger recombination [9]. Since Auger recombination is proportional to the third power of the carrier concentration, its influence was expected to be more severe in GaN-based LDs which operate at a high current density of several kA/cm^2^ [10]. However, few studies have discussed the influence of Auger recombination in GaN-based LDs.

In this paper, we have simulated the carrier recombination behavior in *c*-plane GaN-based LDs and studied the *J_max_* of GaN-based LDs before lasing numerically and experimentally, and the influence of Auger recombination is discussed.

## 2. Experimental

**Figure 1:**
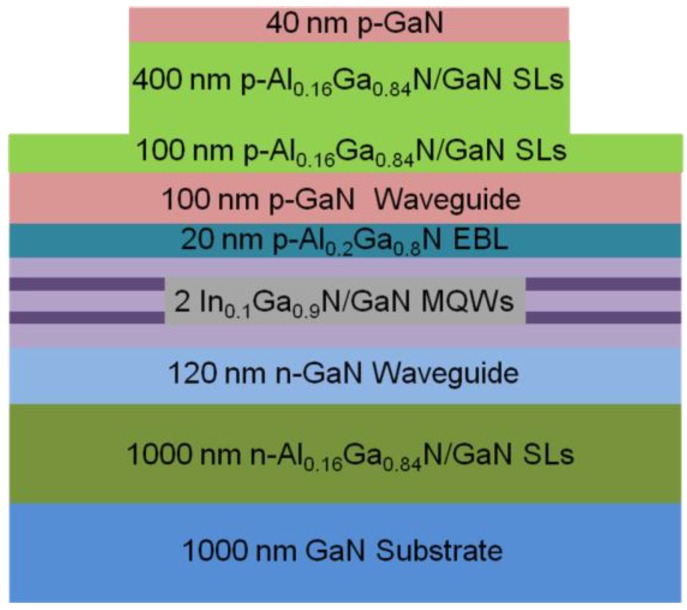
The schematic diagram of *c*-plane GaN-based LDs

The laser structure under study is shown in Figure 1, which is a conventional structure and has been reported in many studies [11,12]. It is composed of a 1000-nm-thick *n*-type GaN layer (Si: 3 × 10^18^ cm^−3^), a 1000-nm-thick *n*-type Al_0.16_Ga_0.84_N/GaN superlattices (SLs) (Si: 3 × 10^18^ cm^−3^), a 120-nm-thick *n*-type GaN lower waveguide layer (Si: 5 × 10^17^ cm^−3^), the quantum-well (QWs) active region, a 20-nm-thick *p*-type Al_0.2_Ga_0.8_N electron blocking layer (EBL) (Mg: 4 × 10^19^ cm^−3^), a 100-nm-thick *p*-type GaN upper waveguide layer (Mg: 1 × 10^19^ cm^−3^), a 500-nm-thick *p*-type Al_0.16_Ga_0.84_N/GaN SLs (Mg: 2 × 10^19^ cm^−3^), and a 40-nm-thick *p*-type GaN contact layer (Mg: 1 × 10^20^ cm^−3^). The QWs consist of two 2.5-nm-thick undoped In_0.1_Ga_0.9_N well layers and three 14-nm-thick *n*-GaN barrier layers (Si: 1 × 10^17^ cm^−3^).

The characteristics of *c*-plane GaN-based LDs are simulated by using a commercial software *LASTIP*, from Crosslight Software Inc., Vancouver, BC, Canada. The simulator is based on finite element analysis of the drift-diffusion model with full Fermi–Dirac statistics for the transport equations as well as for the Poisson equation including the effect of polarization charges in two dimensions [11]. In the simulation, the activation energy of Mg acceptor is taken as 170 meV for GaN [13], which is assumed to increase 3 meV per 1% of Al increment for AlGaN alloy [14]. The width and etching depth of the ridge are chosen as 2 and 0.44 μm, respectively. The cavity length is 600 μm, and the reflectivity values of the front facet and rear facet are 30% and 95%, respectively. The data of refractive indices are taken as the same as those reported in previous studies [11]. The absorption coefficient of Mg-doped GaN depends on doping concentration of Mg as reported in Ref. [14], and the absorption coefficients of Si-doped and undoped GaN are taken as 5 cm^−1^ [15]. For AlGaN, absorption coefficients are taken to be approximately the same as GaN. The built-in interface charges due to spontaneous and piezoelectric polarizations are calculated by the method developed by Fiorentini et al. [16]. A factor of 0.5 of the theoretical built-in interface charges is taken in the simulation due to the compensation by fixed defects and other interface charges. The coefficients of Shockley–Read–Hall (SRH) recombination, spontaneous emission, and Auger recombination are set as 4.2 × 10^7^ s^−1^, 3 × 10^−12^ cm^3^s^−1^, and 4.5 × 10^−31^ cm^−6^s^−1^, respectively, as reported in Ref. [7].

To examine the simulation results, a *c*-plane GaN-based LD was fabricated. The structure is the same as the simulated one. The ridge structure was formed by ion beam etching, and the width and etching depth of the ridge are 2 and 0.44 μm, respectively. The cavity length is 600 μm.

## 3. Results and Discussion

### 3.1. Efficiency Droop Phenomenon

**Figure 2:**
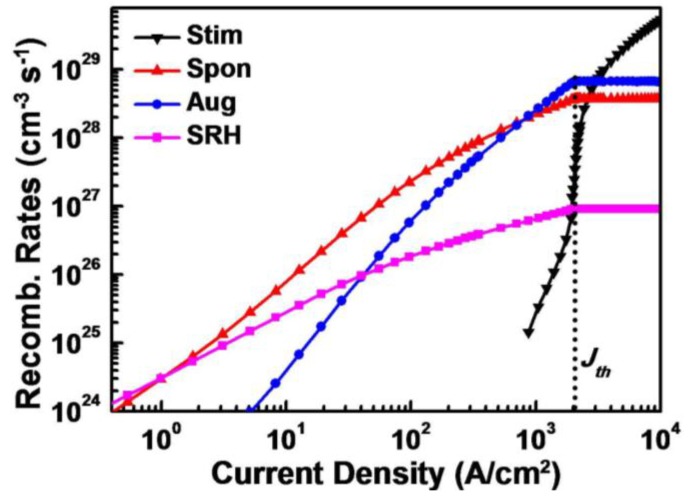
The calculated recombination rates of stimulated emission (black), spontaneous emission (red), Auger recombination (blue), and SRH recombination (magenta) of GaN-based LDs at various injection current densities.

By using the above-mentioned simulator and parameters, we calculated the recombination rates of SRH recombination, spontaneous emission, Auger recombination, and stimulated emission of GaN-based LDs at various injection current levels. As shown in Figure 2, defect-related SRH recombination, which is proportional to the carrier density, dominates the recombination process at a low injection current level. However, it increases very slowly with increasing injection current. Beyond the current density of 1.1 A/cm^2^, spontaneous emission, which depends linearly on the square of carrier density, overtakes the recombination process. Auger recombination, which is proportional to the cube of carrier density, exceeds spontaneous emission at a current density of 830 A/cm^2^. The stimulated emission appears at an injection current density of 875 A/cm^2^. Such manifestation of stimulated emission in InGaN/GaN QWs was also reported by Schwarz [5]. Stimulated emission is a very fast recombination process and increases dramatically with increasing injection current. At the threshold current density (*J_th_*) of 2.1 kA/cm^2^, the carrier density in QWs is clamped, and all recombination rates are saturated except the stimulated emission one. With further increase of injection current, stimulated emissions will continue to raise and dominate the recombination process quickly.

**Figure 3:**
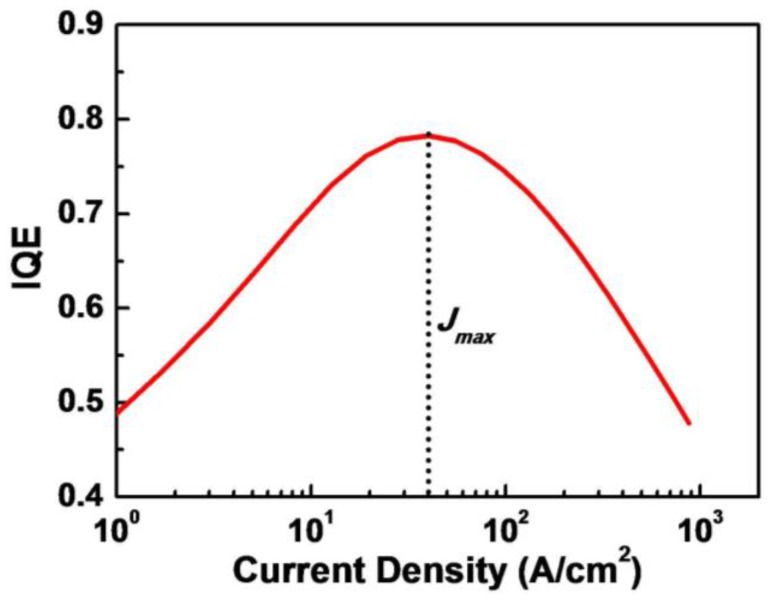
The calculated relationship between IQE and injection current density of GaN-based LDs before lasing.

Simulation results show that no leakage current is observed due to the effective blocking of Al_0.2_Ga_0.8_N EBL (not shown here), which means that all injection current feeds carrier recombination. The IQE of GaN-based LDs before lasing could be calculated as the ratio of spontaneous emission rate over the total recombination rate of SRH recombination, spontaneous emission, and Auger recombination before the appearance of stimulated emission at the current density of 875 A/cm^2^. As shown in Figure 3, the IQE increases with the injection current until it reaches the maximum value of 75.5%. After that, it drops steadily. The current density corresponding to the peak efficiency before lasing, *J_max_*, is nearly 40 A/cm^2^, which is much lower than 133 A/cm^2^ reported by Lutgen et al. [6].

**Figure 4:**
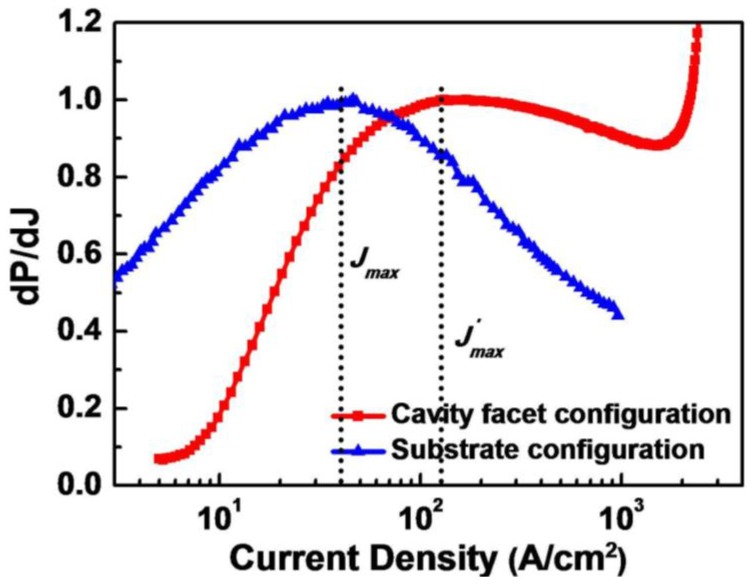
The *dP/dJ–J* curves of a 405 nm GaN-based LD measured from the cavity facet side (red) and the substrate side (blue), respectively.

To find out the reason behind this, we have measured the light output power of the fabricated *c*-plane GaN-based LD at various injection current levels in two different configurations. One is measured from the front cavity facet as reported by Lutgen et al. [6], and the other one is detected from the substrate side. Figure 4 shows the normalized *dP*/*dJ*–*J* curves of the LD measured from both the cavity facet and the substrate side. The normalized *dP*/*dJ*–*J* value is proportional to the IQE, we regard the values of *dP*/*dJ* as the IQE in this paper. Both *dP*/*dJ*–*J* curves show an efficiency droop effect yet with a different *J_max_*. The *J_max_* of 40 A/cm^2^ measured from the substrate side is the same as the simulation result. However, it increases to 130 A/cm^2^ when measured from the cavity facet, which is approximately equal to the reported *J_max_* measured from the cavity facet by Lutgen et al. [6]. Schwarz et al. suggested that stimulated emission may influence the radiative recombination even at a relatively low current density [5], which may result in this difference. Since stimulated emission plays a more and more important role with increasing the injection current, the contribution of stimulated emission to the IQE of GaN-based LDs will rise with increasing injection current, so the *J_max_* would be shifted to a lower value. According to the simulation result, the stimulated emission does not emerge until a current density of 875 A/cm^2^, therefore it cannot explain the difference of *J_max_*, neither does the Fabry–Perot cavity effect.

In fact, the absorption of the QWs should account for the increased *J_max_* when measured from the cavity facet. Due to the good confinement of AlGaN/GaN SLs, most of the light detected from the cavity facet has to travel through the QWs, and will be absorbed by the QWs [17]. At a lowcurrent injection level, the QWs are far from being transparent, and the absorption of the QWs is significant, which makes the normalized *dP*/*dJ* value measured from the cavity facet distinctly lower than that measured from the substrate side. By increasing the injection current, the carrier density in the QWs goes up, and the light absorption of the QWs is reduced. Therefore, the *dP*/*dJ* value measured from the cavity facet increases much faster than that measured from the substrate side until the injection current density reaches the *J_max_* of 130 A/cm^2^. After that, the QWs change from absorption to gain continuously and gradually, the Fabry–Perot cavity effect and stimulated emission start to take over gradually before lasing. Hence, the *dP*/*dJ* value measured from the cavity facet drops very slowly and the efficiency droop phenomenon is less severe than that measured from the substrate side. Therefore, absorption of the QWs shifts the *J_max_* of the GaN-based LD measured from the cavity facet before lasing to a higher value.

The *J_max_* measured from the substrate side is true—i.e., 40 A/cm^2^—which is still higher than that of typical GaN-based LEDs. The lateral spreading of current is supposed to be one of the reasons [18]. In the *p*-AlGaN/GaN SLs, the lateral mobility of holes is much larger than that along the vertical direction [19], the injected holes may spread laterally into the unetched *p*-AlGaN/GaN SLs and *p*-GaN waveguide layer outside the ridge, so the effective width of current injection area is larger than the ridge width, which raises the *J_max_* of GaN-based LDs.

### 3.2. Influence of Auger Recombination

**Figure 5:**
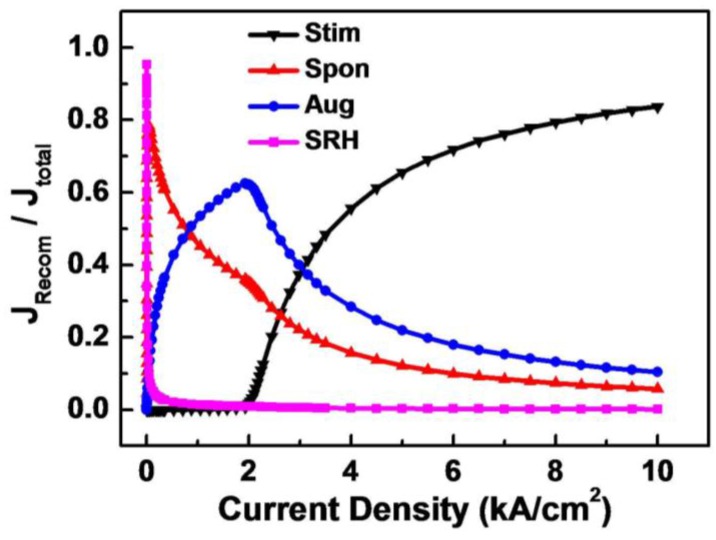
The calculated curves for the ratio of stimulated emission current (black), spontaneous emission current (red), Auger recombination current (blue), and SRH recombination current (magenta) over the total injection current in GaN-based LDs.

In the simulation, the recombination rates of SRH recombination, spontaneous emission, Auger recombination, and stimulated emission of GaN-based LDs at various injection current levels were calculated. By integrating all the recombination rates at various currents, the ratio of SRH recombination rate over the total recombination rate can be obtained, which is equal to the ratio of SRH recombination current over the total injection current as no leakage current exists. The same method could be applied to the recombination current ratios of spontaneous emission, Auger recombination, and stimulated emission. As shown in Figure 5, at the threshold current density of 2.1 kA/cm^2^, the currents supplied to Auger recombination, spontaneous emission, SRH recombination, and stimulated emission are calculated to be 60.9%, 33.8%, 4.5%, and 0.8%, respectively. Most of the injection current feeds Auger recombination which is proportional to the third power of carrier density. Since the carrier density in QWs is clamped after lasing, all recombination rates are saturated except the stimulated emission one. The stimulated emission current increases dramatically with increasing injection current and dominates the injection current very quickly, which makes the ratio of the current supplied to Auger recombination, spontaneous emission, and SRH recombination decrease. At an injection current density of 10 kA/cm^2^, the current supplied to Auger recombination, spontaneous emission, and SRH recombination are reduced to 10.4%, 5.8%, and 0.1%, respectively. The stimulated emission current is increased to 83.7%.

Therefore, Auger recombination influences the threshold current of GaN-based LDs greatly. With increasing injection current, Auger recombination plays a smaller role in GaN-based LDs after lasing. At a high current injection level, the influence of Auger recombination is small, which also applies to spontaneous emission and SRH recombination.

## 4. Conclusions

In summary, the carrier recombination behavior in GaN-based LDs is numerically calculated by using the commercial software *LASTIP*. The efficiency curves were measured from both the cavity facet and the substrate side. Efficiency droop phenomenon in GaN-based LDs was proven theoretically and experimentally before lasing. The *dP*/*dJ* value measured from the cavity facet was modulated by absorption of QWs, which shifts the *J_max_* of GaN-based LDs measured from the cavity facet before lasing to a higher current density. Moreover, the current supplied to various recombinations is calculated. It is found that Auger recombination influences the threshold current greatly, but it only plays a small role at a high current injection level.
